# Several Critical Cell Types, Tissues, and Pathways Are Implicated in Genome-Wide Association Studies for Systemic Lupus Erythematosus

**DOI:** 10.1534/g3.116.027326

**Published:** 2016-03-23

**Authors:** Lu Liu, Xianyong Yin, Leilei Wen, Chao Yang, Yujun Sheng, Yan Lin, Zhengwei Zhu, Changbing Shen, Yinjuan Shi, Yajie Zheng, Sen Yang, Xuejun Zhang, Yong Cui

**Affiliations:** * Institute of Dermatology, Department of Dermatology, The First Affiliated Hospital, Anhui Medical University, Hefei, Anhui Province, 230032, China; † Key Laboratory of Dermatology, Ministry of Education, State Key Lab of Dermatology Incubation Center, Anhui Medical University, Hefei, Anhui Province, 230032, China; ‡ Key Laboratory of Gene Resource Utilization for Complex Diseases, Hefei, Anhui Province, 230032, China; § Collaborative Innovation Center for Complex and Severe Dermatosis, Anhui Medical University, Hefei, Anhui Province, 230032, China; ** Department of Genetics, The Renaissance Computing Institute, University of North Carolina at Chapel Hill, North Carolina 27517; †† Department of Dermatology, China-Japan Friendship Hospital, Beijing, 100029, China

**Keywords:** systemic lupus erythematosus, genome-wide association studies, SNPsea, Genetics of Immunity

## Abstract

We aimed to elucidate the cell types, tissues, and pathways influenced by common variants in systemic lupus erythematosus (SLE). We applied a nonparameter enrichment statistical approach, termed SNPsea, in 181 single nucleotide polymorphisms (SNPs) that have been identified to be associated with the risk of SLE through genome-wide association studies (GWAS) in Eastern Asian and Caucasian populations, to manipulate the critical cell types, tissues, and pathways. In the two most significant cells’ findings (B lymphocytes and CD14+ monocytes), we subjected the GWAS association evidence in the Han Chinese population to an enrichment test of expression quantitative trait locus (QTL) sites and DNase I hypersensitivity, respectively. In both Eastern Asian and Caucasian populations, we observed that the expression level of SLE GWAS implicated genes was significantly elevated in xeroderma pigentosum B cells (*P* ≤ 1.00 × 10^−6^), CD14+ monocytes (*P* ≤ 2.74 × 10^−4^) and CD19+ B cells (*P* ≤ 2.00 × 10^−6^), and plasmacytoid dendritic cells (pDCs) (*P* ≤ 9.00 × 10^−6^). We revealed that the SLE GWAS-associated variants were more likely to reside in expression QTL in B lymphocytes (q_1_/q_0_ = 2.15, *P* = 1.23 × 10^−44^) and DNase I hypersensitivity sites (DHSs) in CD14+ monocytes (q_1_/q_0_ = 1.41, *P* = 0.08). We observed the common variants affected the risk of SLE mostly through by regulating multiple immune system processes and immune response signaling. This study sheds light on several immune cells and responses, as well as the regulatory effect of common variants in the pathogenesis of SLE.

Systemic lupus erythematosus (SLE) is a common autoimmune disease. It is well characterized by the production of a variety of antinuclear antibodies, which then result in a wide spectrum of clinical symptoms in skin, blood, kidney, and lung(Lau *et al.* 2006; Olson *et al.* 2002). Environmental factors and genetic predisposition both contribute to the risk of SLE (Tsokos 2011). In recent years, the genetic basis of SLE has been advanced remarkably, mostly through genome-wide association studies (GWAS) in diverse populations (Remmers *et al.* 2007; Cunninghame Graham *et al.* 2008; Graham *et al.* 2008; International Consortium for Systemic Lupus Erythematosus *et al.* 2008; Kozyrev *et al.* 2008; Hom *et al.* 2008; Musone *et al.* 2008; Nath *et al.* 2008; Gateva *et al.* 2009; Jacob *et al.* 2009; Webb *et al.* 2009; Yang *et al.* 2009b, 2009a; Chang *et al.* 2009; Cunninghame Graham *et al.* 2011; Zhao *et al.* 2011; Luo *et al.* 2011; So *et al.* 2011). Multiple GWAS have been conducted for SLE in Eastern Asian populations, identifying 63 single-nucleotide polymorphisms (SNPs) with genome-wide significant evidence (Zhang *et al.* 2014; Yang *et al.* 2013; Li *et al.* 2013; Sheng *et al.* 2011, 2015; Han *et al.* 2009; Zhang *et al.* 2011; Yu *et al.* 2013; Sun *et al.* 2016). Meanwhile, ∼118 susceptibility SNPs have been established robustly in the Caucasian populations (Graham *et al.* 2008; International Consortium for Systemic Lupus Erythematosus *et al.* 2008; Kozyrev *et al.* 2008; Hom *et al.* 2008; Gateva *et al.* 2009; Lessard *et al.* 2011; Chung *et al.* 2011; Cunninghame Graham *et al.* 2011; Fernando *et al.* 2012; Lessard *et al.* 2012; Lee *et al.* 2012; Martin *et al.* 2013; Armstrong *et al.* 2014; Kariuki *et al.* 2015; Vaughn *et al.* 2014; Demirci *et al.* 2016; Bentham *et al.* 2015; Suarez-Gestal *et al.* 2009). These findings together promote genetic etiology research for SLE. However, the mechanism underlying each variant is still largely unknown. It is usually found that the variants with robust association evidence in complex diseases GWAS are tag SNPs that are linked to the true putative variants, rather than the putative variant itself. In addition, the vast majority of these variants reside in the noncoding genomic region, which therefore poses a great challenge for interpretation and follow-up biological studies (Cui *et al.* 2013). Nevertheless, it is widely indicated that these variants contribute to the risk of diseases mainly through regulating gene expression levels by expression quantitative trait loci (eQTL) effects, or by changing chromatin accessibility, rather altering protein function directly (Edwards *et al.* 2013). In order to disentangle the functional role of each variant in the disease, well-designed biological experiments are warranted. It is strongly believed that each variant contributes to the etiology of disease by affecting a typical tissue and cell through a complicated biological pathway. Hence, in order to dissect the functionality of each GWAS-identified variant, we have to use the most relevant cells and tissues in biological experiments. Nowadays, there are several integrative bioinformatics methods dedicated to identifying the cell types, tissues, and pathways affected by GWAS-implicated susceptibility variants (Weng *et al.* 2011). Some critical cells, tissues, and pathways have been revealed for Crohn disease, rheumatoid arthritis, and multiple sclerosis (Hu *et al.* 2011). Among them, Hu *et al.*(2011) provided the evidence to underpin the key role of transitional B lymphocyte cells in the etiology of SLE.

In the present study, we employed a nonparameter enrichment statistical approach to evaluate the specificity of conditions (*i.e.*, cells, tissues and pathways) in SLE (Slowikowski *et al.* 2014), and tested the enrichment of eQTL sites and DNase I hypersensitivity sites (DHSs) in SLE GWAS common variants.

## Materials and Methods

**Figure 1:**
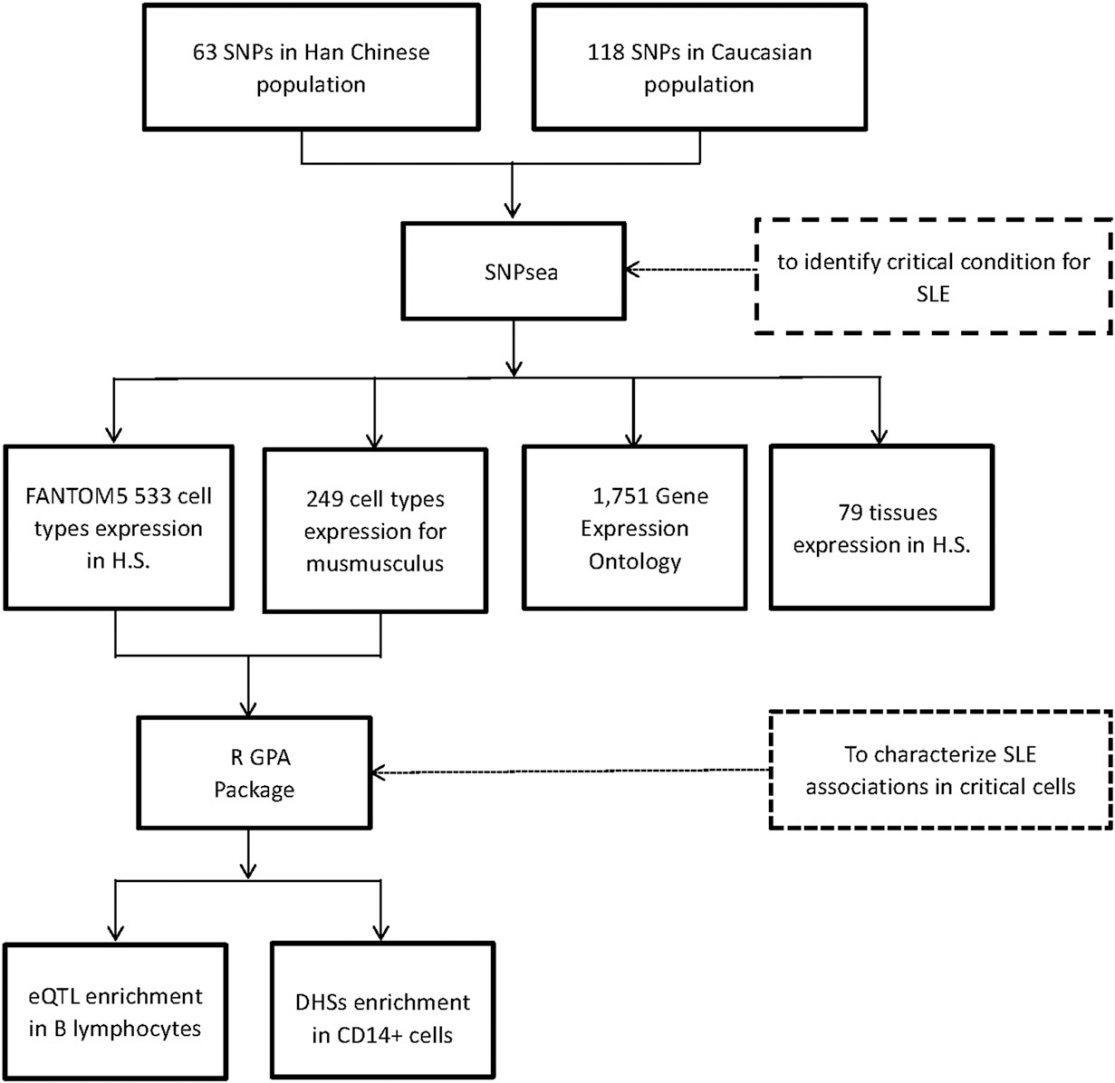
The study design and analytical process diagram. H.S., *Homo sapiens*; eQTL, expression quantitative trait locus; DHSs, DNase I hypersensitive sites.

The study design and analytical process are diagrammed in Figure 1. The enrichment test was implemented in SNPsea (Slowikowski *et al.* 2014). We used 63 and 118 SNPs that had been reported previously with genome-wide evidence in Eastern Asian and Caucasian populations, respectively, as input (Supplemental Material, Table S1). It was noted that 13 of the 118 SNPs from Caucasian populations reside in the *HLA* region. In SNPsea, we identified genes implicated by each input SNP using linkage disequilibrium (LD) information in the CHB or CEU reference panel, respectively, from the 1000 Genomes Project. To include all potential genes, we extended the SNP interval to the nearest recombination hotspots with recombination rate > 3 cM/Mb (HapMap 3) (Myers *et al.* 2005). If a SNP interval overlapped with no gene intervals, we extended the SNP interval to 10,000 base pairs. If one unified gene was implicated by at least two different SNPs, we merged these SNPs into one single locus. Usually, multiple genes would be implicated in each locus. But we assumed that there would be only one single gene truly associated with SLE in each locus. We normalized the expression of each gene through dividing its expression value in a specific condition (*i.e.*, the cell types, tissues, and pathways) by the L2 norm of gene expression values for that gene in all different conditions, thus we would get an expression matrix with values between zero and one, which was used to indicate the specificity of the gene to a condition. For each condition, we ranked the normalized expression values in descending order, and divided them by the number of implicated genes. As a result, we got a score between zero and one, with smaller values representing a higher specificity for a given condition (Hu *et al.* 2011). We assigned the lowest score for each locus. For each condition, we used a sampling approach to build a null SNP set, and then calculated an empirical *P*-value through comparing its gene expression distribution with that of the SLE GWAS SNP set. This empirical *P*-value was used to indicate the tail probability of observing a condition-specificity score greater than, or equal to, the sum across all SNPs (Slowikowski *et al.* 2014). The significance threshold was defined by Bonferroni correction with the number of conditions used in each test. We applied 533 cell types expression matrix for *Homo sapiens* from FANTOM5 (Bonferroni *P* threshold = 0.05/533), 249 cell types expression matrix for *Mus musculus* (GEO dataset: GSE15907, Bonferroni *P* threshold = 0.05/249), 79 tissues expression matrix for *H. sapiens* (Bonferroni *P* threshold = 0.05/79) (Su *et al.* 2004), and 1751 gene expression ontology (GO) matrix (data-version: 2013-06-29, CVS revision: 9700, Bonferroni *P* threshold = 0.05/1751), to test the specificity of cell types, tissues and pathways, respectively.

The role of B lymphoblastoid cells in the pathogenesis of SLE is well known. Our results in the SNPsea test also identified the significant evidence of B lympholastoid cells in both Eastern Asian and Caucasian populations. In addition, this test revealed the most significant evidence for CD14+ monocytes in the pathophysiology of SLE. In an attempt to unravel which functional part the SLE associated variant most likely resides in, we incorporated the SLE GWAS summary statistics in Han Chinese population, eQTL from B lymphoblastoid cells and DHSs functional genomics data from CD14+ cells. We retrieved the GWAS summary statistics for SLE described preciously in the Han Chinese (Han *et al.* 2009). These contained the association evidence (single variant *P* value) for 494,559 autosomal variants. The eQTL results for whole blood B lymphocytes in 5311 Caucasian individuals were freely accessed (Westra *et al.* 2013). In total, there were 923,021 and 4733 SNPs annotated to be *cis*-eQTL and *trans*-eQTL sites, respectively, in whole blood B lymphocytes. The DHSs for CD14+ primary cells were downloaded from the Encyclopedia of DNA Elements (ENCODE) consortium database (accession: ENCFF001SRV), in which there were 159,457 DHSs identified for CD14+ monocytes. Each of the 494,559 autosomal variants SNP in SLE GWAS was then annotated by whether it resided in the DHSs in CD14+ monocytes, or the eQTL site in whole blood B lymphocytes, respectively. The enrichment of DHSs and eQTL effect were tested through genetic analysis incorporating pleiotropy and annotation (GPA) packages in R 3.12 (Chung *et al.* 2014). The enrichment size of DHS or eQTL site was characterized by estimating the enrichment fold (*q*_1_/*q*_0_), which was calculated as odds of proportional annotated by CD14+ DHS, or whole blood lymphocytes, eQTL effect in non-null- and null-associated SNPs (Chung *et al.* 2014). *q*_1_ and Q_0_ were defined as the probability of being annotated as DHS or eQTL sites for non-null- and null-associated SNPs, respectively. The statistical significance for the enrichment effect was evaluated by likelihood ratio test. The maximum iteration was set to 10,000 in the GPA package.

### Data availability

The authors state that all data necessary for confirming the conclusions presented in the article are represented fully within the article.

## Results

**Figure 2:**
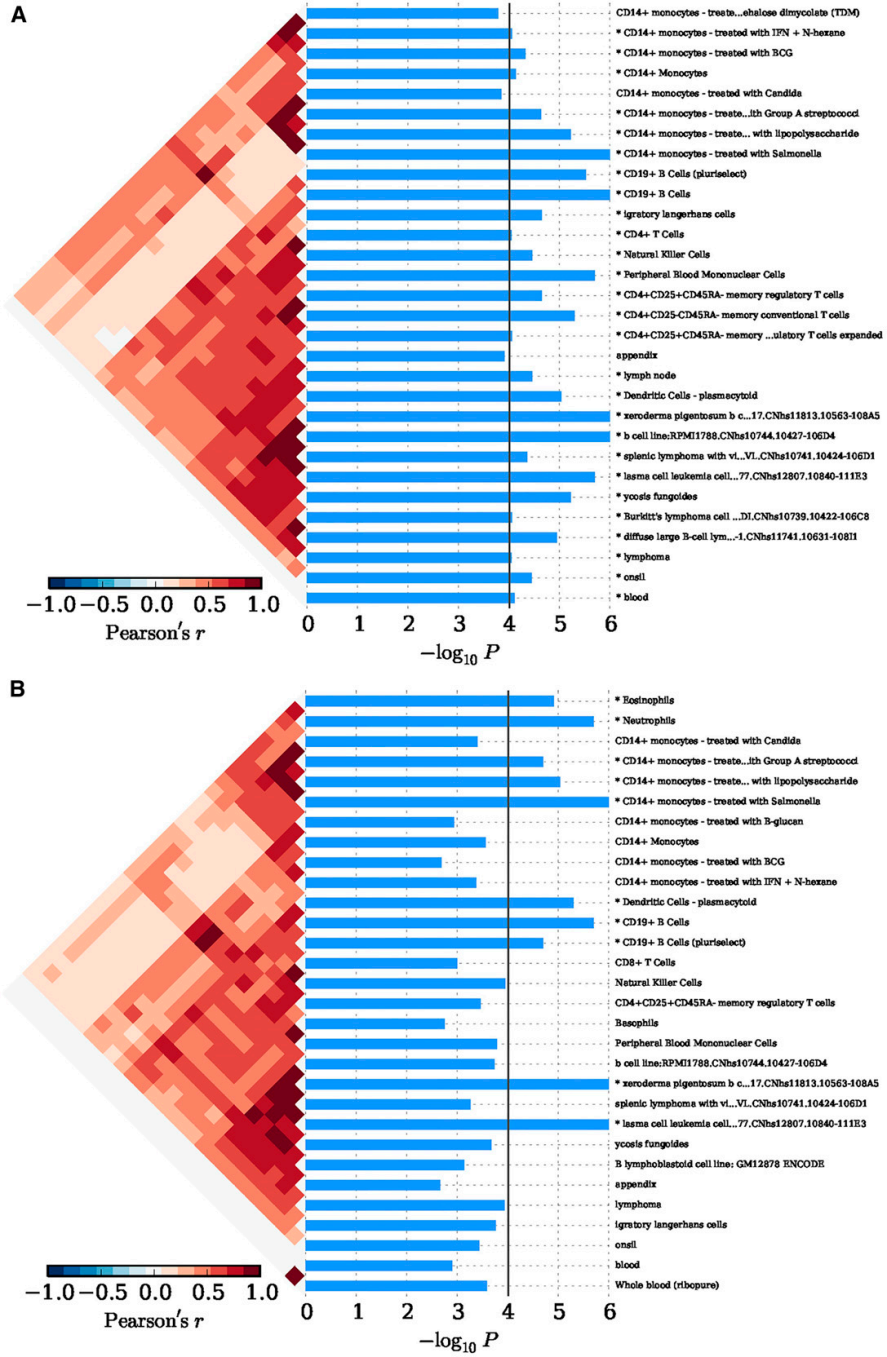
The cell enrichment of SLE implicated genes, with 533 cell types expression matrix in *H. sapiens*. (A) Cell enrichment by 63 SNPs in an Eastern Asian population. (B) Cell enrichment by 118 SNPs in a Caucasian population. The bottom indicates the log transformed *P* value. The vertical line indicates the Bonferroni-corrected significance criteria (*P* ≤ 9.38 × 10^−5^). The cell types are listed on the right.

**Figure 3:**
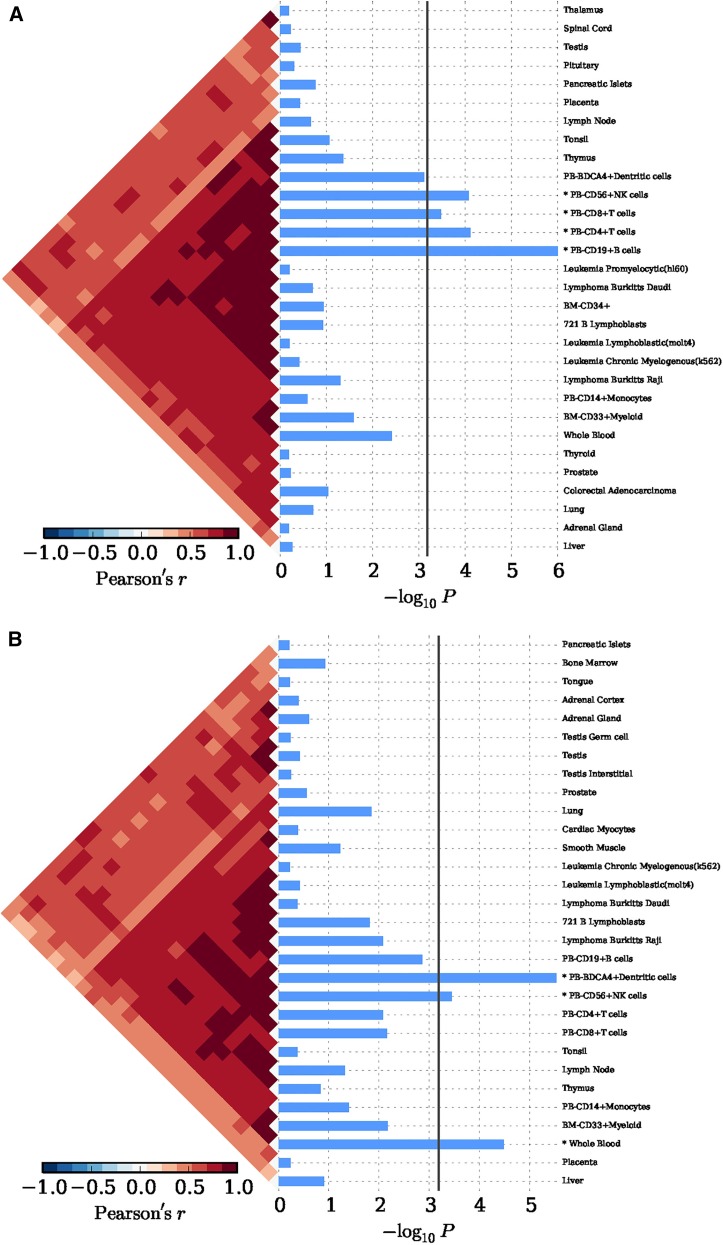
The tissue enrichment of SLE implicated genes with 79 tissues expression matrix in *H. sapiens*. (A) Tissue enrichment by 63 SNPs in an Eastern Asian population. (B) Tissue enrichment by 118 SNPs in a Caucasian population. The bottom indicates the log transformed *P* value. The vertical line indicated the Bonferroni-corrected significance criteria (*P* ≤ 6.33 × 10^−4^). The tissues names are listed in the right.

In Eastern Asian populations, it has been shown that the expression of the implicated genes were significantly enriched in 19 types of cells, for example, xeroderma pigentosum B cells (*P* = 1.00 × 10^−6^), CD14+ monocytes (*P* = 7.33 × 10^−5^), CD19+ B cells (*P* = 1.00 × 10^−6^), plasmacytoid dendritic cells (pDCs) (*P* = 9.00 × 10^−6^), and CD4+CD25+CD45RA– memory regulatory T cells (*P* = 2.20 × 10^−5^) (Figure 2A and Table S2). And significant evidence regarding B cells (*e.g.*, B.T1.sp, B.T2.sp, B.T3.sp, B.Fo.sp, B.MZ.Sp, B1a.sp, B.FrF.BM, and B1a.pc etc.) was achieved consistently with using 249 cell types expression matrix for *Mus musculus* (*P* ≤ 4.88 × 10^−5^; Figure S1A and Table S3). In peripheral blood, we achieved corrected significance at CD19+ B cells (*P* = 1.00 × 10^−6^), CD4+ T cells (*P* = 7.57 × 10^−5^), CD56+NK cells (*P* = 8.30 × 10^−5^) (Figure 3A and Table S4). We only observed GO:0002684 positive regulation of immune system process (*P* = 1.60×10^-5^) was significantly elevated, although a series of the immune response pathways achieved moderate significance (Figure S2A and Table S5). In Caucasian populations, the cell enrichment test also indicated that xeroderma pigentosum B cells (*P* = 1.00 × 10^−6^), CD14+ monocytes (*P* = 1.00 × 10^−6^), CD19+ B cells (*P* = 2.00 × 10^−6^), and plasmacytoid dendritic cells (pDCs) (*P* = 5.00 × 10^−6^) were significantly activated (Figure 2B and Table S2). Likewise, significant evidence for B cells (*e.g.*, B.T2.sp and B.T3.sp) was also achieved, consistent with using 249 cell types expression matrix for *Mus musculus* (*P* ≤ 6.23 × 10^−5^; Figure S1B and Table S3). In peripheral blood, we also discovered corrected significance at BDCA4+ dentritic cells (*P* = 3.00 × 10^−6^) and CD56+NK cells (*P* = 3.62 × 10^−4^) (Figure 3B and Table S4). The GO pathway enrichment test showed that 38 GO pathways relevant to immune process, cytokine production and cell activation (*P* ≤ 1.90 × 10^−5^) were significantly regulated (Figure S2B and Table S5).

In order to eliminate the possible influence of the *HLA* region on our findings in Caucasian populations, we removed the 13 SNPs within the *HLA* region, and conducted the condition enrichment analysis in the SNPsea. Eventually, we achieved similar and consistent results to those with the *HLA* region variants. The findings indicate that xeroderma pigentosum B cells (*P* = 1.00 × 10^−6^), CD14+ monocytes (*P* = 1.00 × 10^−6^), CD19+ B cells (*P* = 1.00 × 10^−6^), plasmacytoid dendritic cells (pDCs) (*P* = 6.10 × 10^−5^) remained significantly involved in the SLE (Figure S3 and Table S2). Meanwhile, we achieved significant and consistent evidence for B cells (*e.g.*, B.T2.sp, B.T3.sp, CD19+ Control, etc.) using 249 cell types expression matrix for *Mus musculus* (*P* < 6.59 × 10^−5^; Figure S1C and Table S3), and for BDCA4+ dentritic cells in peripheral blood (*P* = 1.00 × 10^−6^; Figure S4 and Table S4). We identified that the multiple immune response pathways remained significantly activated even without the HLA region (Figure 2SC and Table S5).

In our SLE GWAS results in the Han Chinese population, of the 494,559 common variants, 110,893 and 16,981 SNPs were annotated as eQTL site and DHS in human whole blood B lymphocytes and CD14+ monocytes, respectively. We observed a significant enrichment of eQTL effect in the SLE GWAS association evidences (*q*_1_/*q*_0_ = 2.15, *P* = 1.23 × 10^−44^). And we found an enrichment effect of DHS in CD14+ monocytes (*q*_1_/*q*_0_ = 1.41), and this effect achieved approximately nominal significance (*P* = 0.08).

## Discussion

In the past decades, the genetic susceptibility of SLE has been advanced dramatically through GWAS in diverse populations. However, the exact mechanism for each individual variant is still unclear. In order to ultimately dissect the mechanism, biological experiments must be carefully conducted in the most relevant pathogenic cell types and tissues. In the present study, we observed that several immune cells (*e.g.*, B cells, monocytes, and pDCs), and several immune regulating signaling pathways, were significantly activated in the pathogenesis of SLE in both Eastern Asian and Caucasian populations. Our findings indicated that common SLE-associated genetic variants are more likely to reside in eQTL sites and DHSs in whole blood B lymphocytes and CD14+ monocytes, respectively. This result will help the identification of SLE susceptibility variants in future studies.

SLE is a typical autoimmune disease. One of the remarkable hallmarks of SLE is the generation of various immunological abnormalities (Tsokos 2011). It is well accepted that B cells play an important role in its mechanism (Chan *et al.* 1999). Our study underpins the critical role of B cells, especially CD19+ B cells, in the etiology of SLE. In a preliminary study, patients with SLE and secondary antiphospholipid syndrome (APS) showed depletion of CD3-CD19+ B cells, and their decreasing number correlated with the severity of SLE (Dal Ben *et al.* 2014). Using the SLE GWAS summary statistics, we discovered that the associated variants were significantly likely to confer an eQTL effect in whole blood B cells. The results not only confirm the critical role of B cells in the pathology of SLE, but also indicate that the genetic common variants probably contribute to the risk of SLE, mainly by regulating gene expression levels through eQTL effects. It has been broadly implicated that GWAS findings in several common diseases are enriched in eQTL sites (Nicolae *et al.* 2010). In addition, we revealed that the expression level of SLE GWAS implicated genes was significantly elevated in CD14+ monocytes. CD14+ monocytes have been demonstrated to impair phagocytosis of autologous apoptotic polymorphonuclear leukocytes (PMNs) (Mikolajczyk *et al.* 2014). Zhu *et al.* (2014) detected a series of severe disease clinical manifestations and laboratory features in SLE patients [*e.g.*, presence of autoantibodies, 24-hr proteinuria excretion or systemic lupus erythematosus disease activity index (SLEDAI) ≥ 10] were associated with the decreased mAxl expression on CD14+ monocytes. Axl, which is responsible for clearance of apoptotic cells and immune homeostasis maintenance, is a transmembrane receptor tyrosine kinase (RTK) expressed on the surface of monocytes (mAxl) (Seitz *et al.* 2007). Our study revealed that SLE GWAS association evidence was more likely to be enriched in the DHSs in CD14+ monocytes. DHSs are considered as the mark of transcriptionally active regions in the genome, and have cell specificity (Thurman *et al.* 2012). The finding of enrichment of DHSs in GWAS has been found in several other common diseases (Disanto *et al.* 2014; Maurano *et al.* 2012; Handel *et al.* 2013; Chen *et al.* 2015). Additionally, it is particularly noteworthy that we detected pDCs playing a pivotal role in SLE. Studies using various experimental lupus models have revealed that pDCs play an indispensable role in stimulating autoantibody response and facilitate lupus progression, which bolsters the rationale of targeting pDCs to alleviating SLE (Baccala *et al.* 2013; Sisirak *et al.* 2014; Rowland *et al.* 2014; Davison and Jorgensen 2015). Patients with SLE frequently have aberrant expression of genes that are stimulated by type 1 interferons (IFN-α, IFN-β, IFN-ω, IFN-τ, and IFN-I), a family of pluripotent cytokines that are important for antiviral immune response, and this expression profile is correlated with anti-dsDNA antibody levels and disease severity (Bennett *et al.* 2003; Banchereau and Pascual 2006). In SLE patients, pDCs are believed to be a major cellular source of IFN-I, primarily because they readily produce IFN-I when exposed to SLE immune complexes or other lupus-related, nucleic acid-containing compounds (Gilliet *et al.* 2008; Colonna *et al.* 2004; Reizis *et al.* 2011). In the present study, we also detect that Treg cells and NK cells are moderately activated, and confirm the role of these types of cells in the etiology of SLE. Regulatory T (Treg) cells are a subset of CD4+ T cells (Ohl and Tenbrock 2014). So far, it has been found that SLE may occur in connection with reduced numbers or impaired function of circulating Treg cells (Ohl and Tenbrock 2014). Only a minority of human CD4+ T cells expressing the highest levels of CD25 (termed as CD4+CD25+ T cells) maintains self-tolerance by suppressing autoreactive lymphocytes (Ohl and Tenbrock 2014). Seddiki *et al.* (2006) found that CD45RA+ cells are members of the natural TREG lineage. To date, the literature has disregarded NK cells as relevant modulators in the pathogenesis of SLE, and they are rarely observed in SLE, but show dysfunction in patients with active SLE (Spada *et al.* 2015). Last, but not the least, it is notable that the Xeroderma pigentosum (XP) B cell line has been revealed in our study, although there is currently no evidence available linking XP B cells with SLE. It is well known that XP is an autosomal recessive disease, and shares the typical performance of solar sensitivity with SLE patients (Tsokos 2011; Yang *et al.* 2015), which hence makes us believe that one unique mechanism potentially accounts partially for these two diseases. However, further studies are needed to elucidate the exact mechanism. Recently, Sun *et al.* (2016) suggested that certain genes implicated by SLE GWAS SNPs in Eastern Asian samples are significantly expressed in multiple immune cell types (such as XP B cells, CD19+ B cells, CD4+ T cells, and NK cells). In the present study, we implicated these cells types in either Eastern Asian or Caucasian populations. A comparison of the results suggests the important roles of these cell types in the pathogenesis of SLE. Our study underpins the key role of the immune regulating signaling pathways in the pathogenesis of SLE. By analyzing 17 alleles attaining an extremely high bar of statistical significance in the first round of GWAS, investigators demonstrated an important role for several pathways contributing to SLE susceptibility, including B-cell signaling and development, signaling through toll-like receptors 7 and 9, and neutrophil function (Graham *et al.* 2009).

Although we found many similar results between Caucasian and Eastern Asian populations, some differences were also observed. For example, we detected spleen lymphocytes with villous lymphocytes cell line (*P* = 4.27 × 10^−5^) only in Eastern Asian populations. We speculate that this is caused by the relatively smaller number of GWAS findings used in the Eastern Asian population, which leads to the limited number of implicated genes in Eastern Asian populations in our study, as well as ethnical heterogeneity because most of the gene expression matrices used in our study come from Caucasian populations. We note that the role of spleen lymphocytes in SLE has become well recognized. For example, Hu *et al.* (2011) tested the enrichment of SLE-implicated genes within the 223 expression profiles, and revealed transitional splenic B cells. The pathogenic nature of spleen lymphocytes is also supported by mouse models, and by demonstration of the efficacious nature of spleen cells from lupus-prone MRL/lpr mice targeted therapies (Huang *et al.* 2014; Fleischer *et al.* 2014).

There are several weaknesses in our study. First, the condition expression matrices used in our study are less comprehensive and lack population, tissue and cell specificity. For example, the public data we used to generate these results was mostly from Caucasian ancestry. Second, the enrichment of DHSs in CD14+ monocytes did not achieve significance. We assume this is due to the incomplete annotation of DHSs in CD14+ cells. Third, the number of genes implicated with each susceptibility variants relies on the LD information used in the SNPsea algorithm. Thus, more complete LD information will help improve the accuracy in future.

In summary, we implicated several immune cells and immune systems in the pathogenesis of SLE. The findings will help guide the design of future biological experiments for SLE.

## 

## Supplementary Material

Supplemental Material
