# Supplementary material for: Several Critical Cell Types, Tissues, and Pathways Are Implicated in Genome-Wide Association Studies for Systemic Lupus Erythematosus
Source: G3 (Bethesda). 2016 Mar 23;6(6):1503–11. doi: 10.1534/g3.116.027326 (PMC4889647; doi:10.1534/g3.116.027326)
Supplement: Supplemental Material [file supp_6_6_1503__index.html]

Several Critical Cell Types, Tissues, and Pathways Are Implicated in Genome-Wide Association Studies for Systemic Lupus Erythematosus — Supplemental Material 

# Several Critical Cell Types, Tissues, and Pathways Are Implicated in Genome-Wide Association Studies for Systemic Lupus Erythematosus

## Supplemental Material for Liu *et al.*, 2016

**Files in this Data Supplement:**

- Figure S1 - (A) The cell enrichment of SLE genes implicated by 63 SNPs in Eastern Asian population within 249 cell types expression matrix for mus musculus. (B) The cell enrichment of SLE implicated genes by 118 SNPs in Caucasian population within 249 cell types expression matrix for mus musculus. (C) The cell enrichment of SLE implicated gene by 105 SNPs without HLA region SNPs in Caucasian population within 249 cell types expression matrix for mus musculus. (.pdf, 138 KB)
- Figure S2 - (A) The pathway enrichment of SLE genes implicated by 63 SNPs in Eastern Asian population within 1,751 Gene Expression Ontology matrix. (B) The pathway enrichment of SLE implicated genes by 118 SNPs in Caucasian population within 1,751 Gene Expression Ontology matrix. (C) The pathway enrichment of SLE implicated gene by 105 SNPs, without HLA region SNPs in Caucasian population within 1,751 Gene Expression Ontology matrix. (.pdf, 509 KB)
- Figure S3 - The cell enrichment of SLE implicated genes by 105 SNPs, without HLA region SNPs in Caucasian population within 533 cell types expression matrix in homo-sapiens. (.pdf, 175 KB)
- Figure S4 - The tissue enrichment of SLE implicated gene by 105 SNPs without HLA region SNPs in Caucasian population within 79 tissues expression matrix for homo-sapiens. (.pdf, 52 KB)
- Table S1 - The 181 input SNPs with genome-wide significance for Systemic lupus erythematosus. (.pdf, 221 KB)
- Table S2 - The cell enrichment of SLE implicated genes within 533 cell types expression matrix in Homo sapiens. (.pdf, 84 KB)
- Table S3 - The cell enrichment of SLE implicated genes within 249 cell types expression matrix for mus musculus. (.pdf, 76 KB)
- Table S4 - The tissue enrichment of SLE implicated genes within 79 tissues expression matrix in homo-sapiens. (.pdf, 76 KB)
- Table S5 - The pathway enrichment of SLE implicated genes within 1,751 Gene Expression Ontology matrix. (.pdf, 90 KB)
