# Supplementary material for: Several Critical Cell Types, Tissues, and Pathways Are Implicated in Genome-Wide Association Studies for Systemic Lupus Erythematosus
Source: G3 (Bethesda). 2016 Mar 23;6(6):1503–11. doi: 10.1534/g3.116.027326 (PMC4889647; doi:10.1534/g3.116.027326)

Figure S2A. The pathway enrichment of SLE genes implicated by 63 SNPs in Eastern Asian population within 1,751 Gene Expression Ontology matrix. *The bottom indicates the log transformed p value. The vertical line indicated the Bonferroni-corrected significance criteria. The pathways codes and names are listed in the right.*

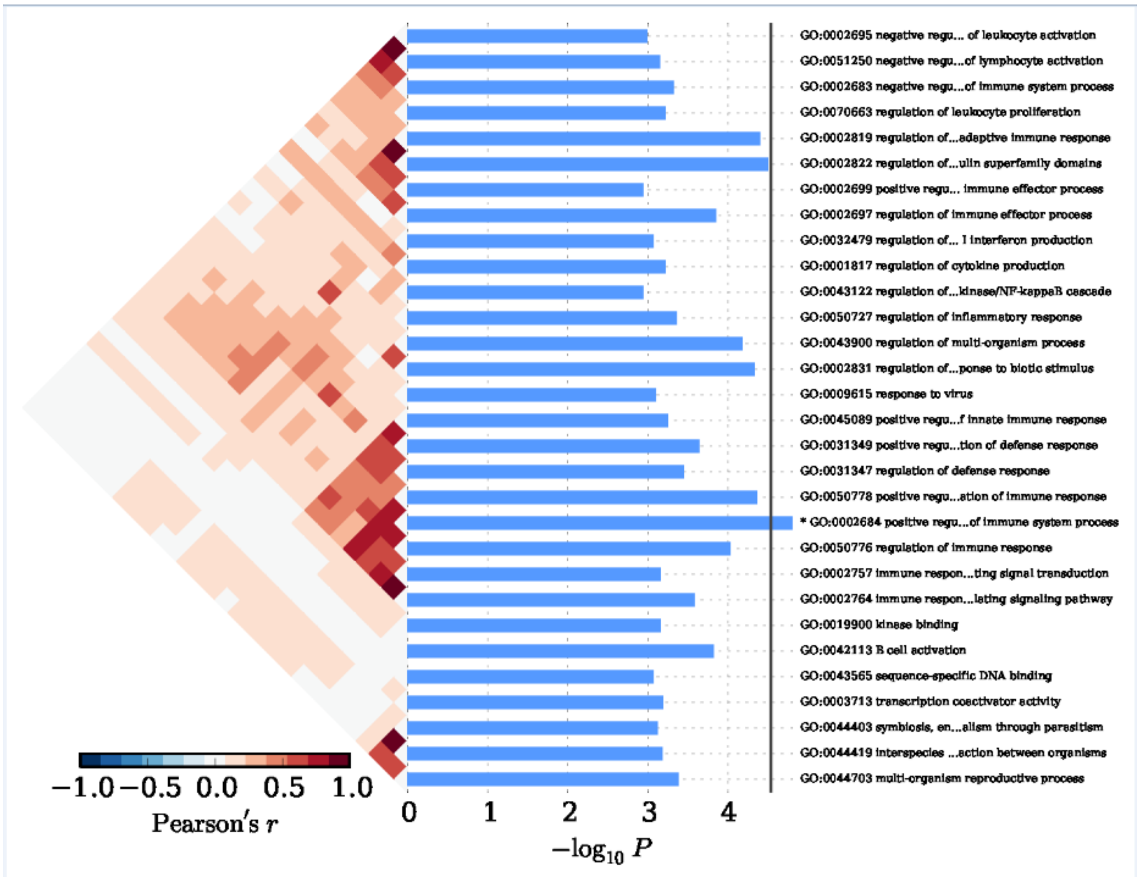

Figure S2B. The pathway enrichment of SLE implicated genes by 118 SNPs in Caucasian population within 1,751 Gene Expression Ontology matrix. *The bottom indicates the log transformed p value. The vertical line indicated the Bonferroni-corrected significance criteria. The pathways codes and names are listed in the right.*

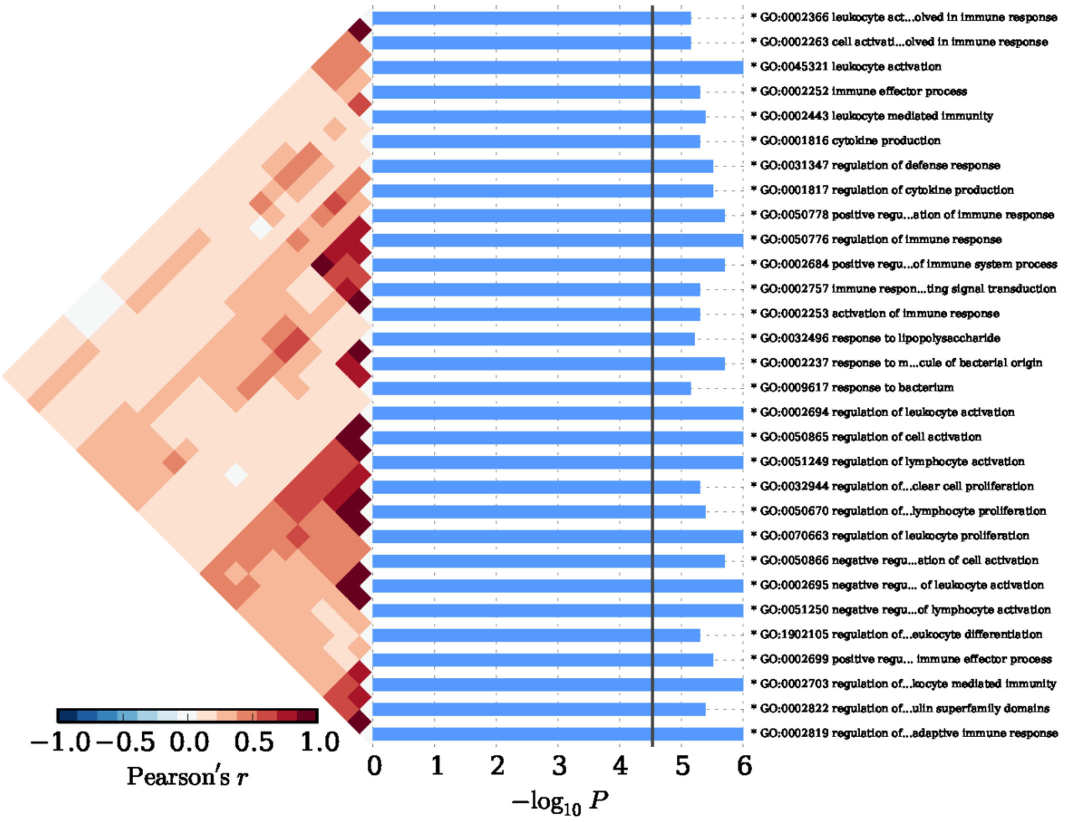

Figure S2C. The pathway enrichment of SLE implicated gene by 105 SNPs, without HLA region SNPs in Caucasian population within 1,751 Gene Expression Ontology matrix. *The bottom indicates the log transformed p value. The vertical line indicated the Bonferroni-corrected significance criteria. The pathways codes and names are listed in the right.*

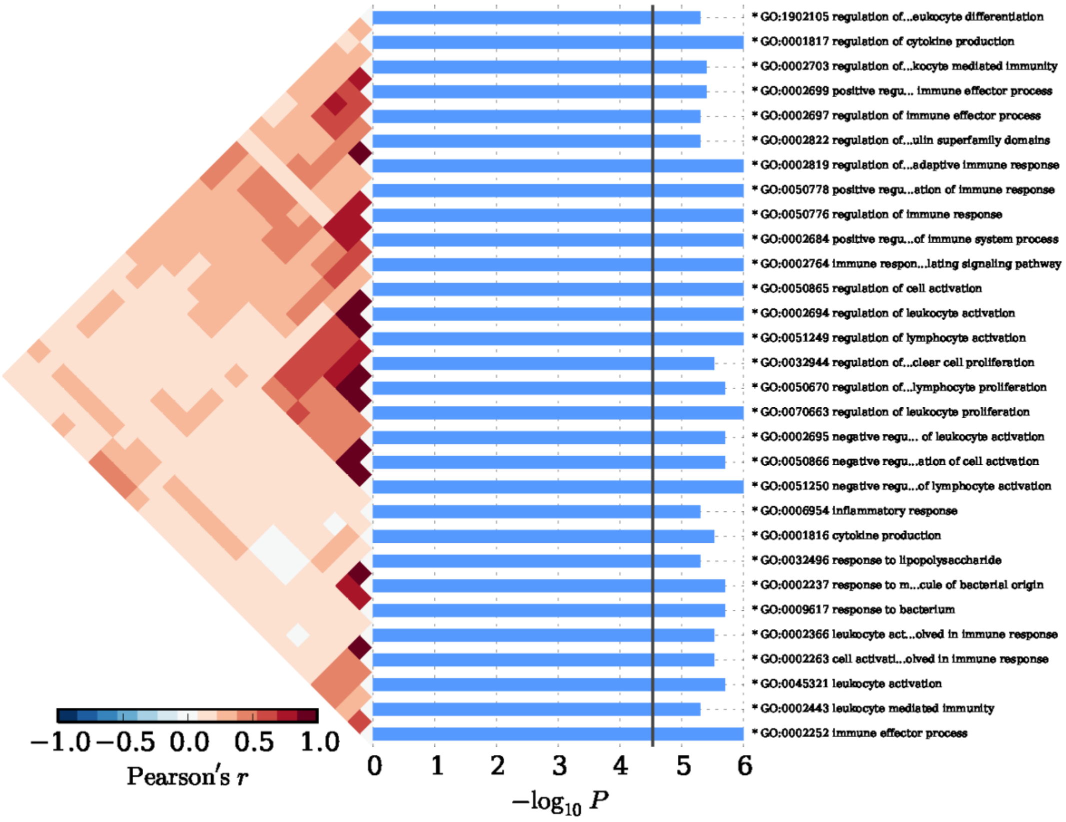

Supplement: Supplemental Material [file supp_g3.116.027326_FigureS2.pdf]
