# Supplementary material for: Several Critical Cell Types, Tissues, and Pathways Are Implicated in Genome-Wide Association Studies for Systemic Lupus Erythematosus
Source: G3 (Bethesda). 2016 Mar 23;6(6):1503–11. doi: 10.1534/g3.116.027326 (PMC4889647; doi:10.1534/g3.116.027326)

Figure S3. The cell enrichment of SLE implicated genes by 105 SNPs, without HLA region SNPs in Caucasian population within 533 cell types expression matrix in homo-sapiens. *The bottom indicates the log transformed p value. The vertical line indicates the Bonferroni-corrected significance criteria. The cell types are listed in the right.*

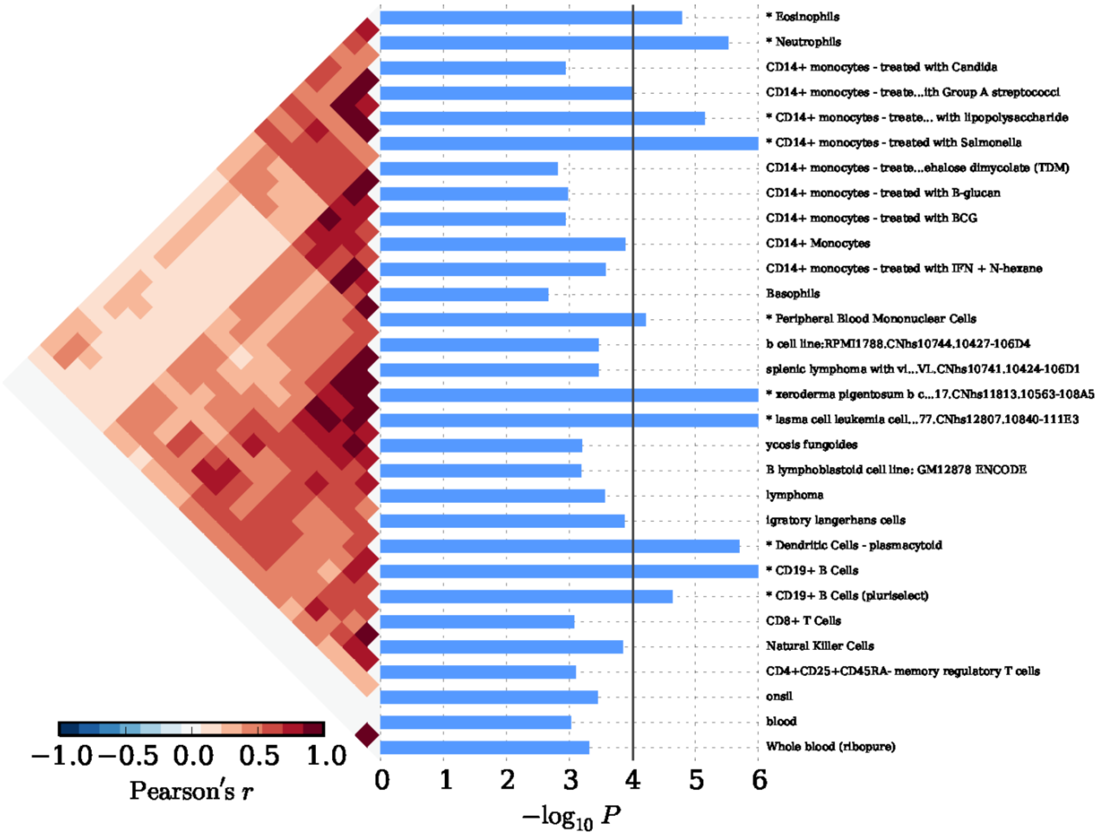

Supplement: Supplemental Material [file supp_g3.116.027326_FigureS3.pdf]
