# Supplementary material for: Several Critical Cell Types, Tissues, and Pathways Are Implicated in Genome-Wide Association Studies for Systemic Lupus Erythematosus
Source: G3 (Bethesda). 2016 Mar 23;6(6):1503–11. doi: 10.1534/g3.116.027326 (PMC4889647; doi:10.1534/g3.116.027326)

Figure S4. The tissue enrichment of SLE implicated gene by 105 SNPs without HLA region SNPs in Caucasian population within 79 tissues expression matrix for homo-sapiens. *The bottom indicates the log transformed p value. The vertical line indicated the Bonferroni-corrected significance criteria. The tissues names are listed in the right.*

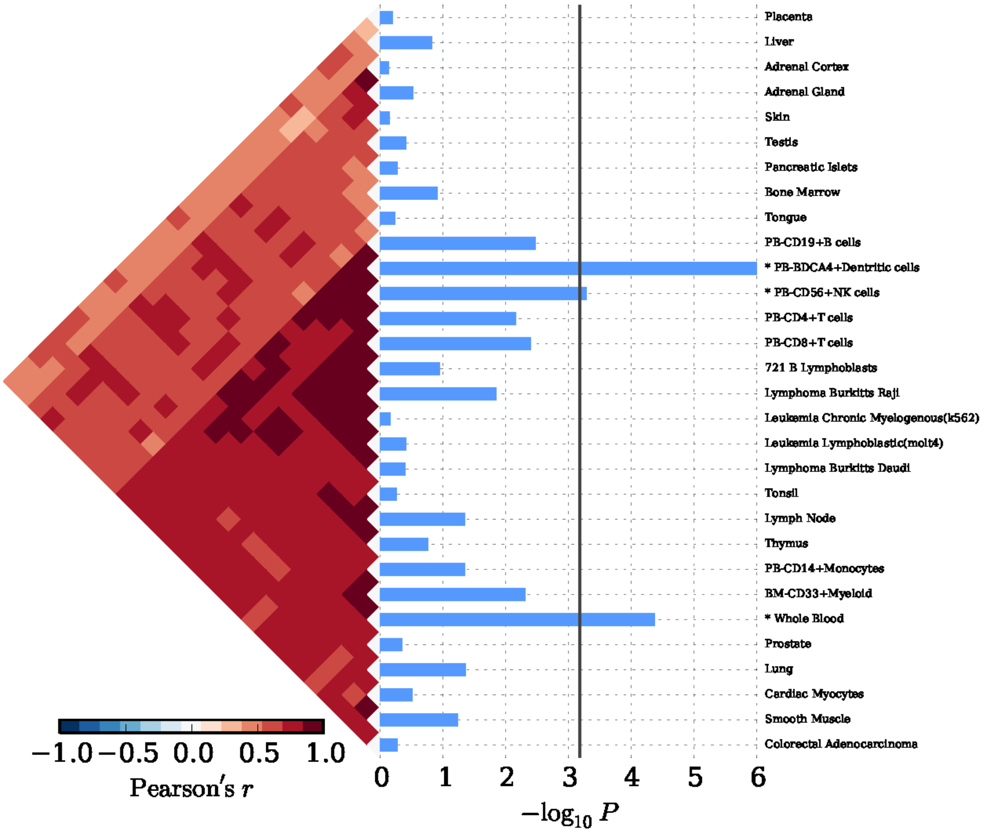

Supplement: Supplemental Material [file supp_g3.116.027326_FigureS4.pdf]
