# Supplementary material for: Several Critical Cell Types, Tissues, and Pathways Are Implicated in Genome-Wide Association Studies for Systemic Lupus Erythematosus
Source: G3 (Bethesda). 2016 Mar 23;6(6):1503–11. doi: 10.1534/g3.116.027326 (PMC4889647; doi:10.1534/g3.116.027326)
Supplement: Supplemental Material [file supp_g3.116.027326_TableS1.pdf]

Table S1. The 181 input SNPs with genome-wide significance for Systemic lupus erythematosus. *Pop: population, EAS: Eastern Asian population; CEU: Caucasian population; SNP: single-nucleotide polymorphism; chr: chromosome; P-value$_{combined}$: The associated p value in the combined samples in previous papers; Ref.: reference.*

**Pop**	**SNP**	**position**	**Chr**	**P-value$_{combined}$**	**Ref**
EAS	rs1234315	173178463	1q25.1	2.34×10^{-26}	(Han et al. 2009)
EAS	rs2205960	173191475	1q25.1	2.53×10^{-32}	(Han et al. 2009)
EAS	rs13385731	33701890	2p22.3	1.25×10^{-15}	(Han et al. 2009)
EAS	rs7574865	191964633	2q32.3	5.17×10^{-42}	(Han et al. 2009)
EAS	rs2230926	138196066	6q23.3	1.37×10^{-17}	(Han et al. 2009)
EAS	rs4917014	50305863	7p12.2	2.75×10^{-23}	(Han et al. 2009)
EAS	rs1167796	75173180	7q11.23	2.12×10^{-08}	(Han et al. 2009)
EAS	rs4728142	128573967	7q32.1	8.14×10^{-19}	(Han et al. 2009)
EAS	rs7812879	11340181	8p23.1	2.09×10^{-24}	(Han et al. 2009)
EAS	rs2618479	11355821	8p23.1	5.26×10^{-21}	(Han et al. 2009)
EAS	rs2248932	11391650	8p23.1	1.63×10^{-21}	(Han et al. 2009)
EAS	rs1913517	50119054	10q11.22	7.22×10^{-12}	(Han et al. 2009)
EAS	rs4639966	118573519	11q23.3	1.25×10^{-16}	(Han et al. 2009)
EAS	rs6590330	128311059	11q24.3	1.77×10^{-25}	(Han et al. 2009)
EAS	rs10847697	129299385	12q24.32	3.54×10^{-11}	(Han et al. 2009)
EAS	rs1385374	129300694	12q24.32	1.77×10^{-11}	(Han et al. 2009)
EAS	rs7197475	30642867	16p11.2	2.77×10^{-08}	(Han et al. 2009)
EAS	rs463426	21809185	22q11.21	1.48×10^{-16}	(Han et al. 2009)
EAS	rs131654	21917190	22q11.21	2.99×10^{-16}	(Han et al. 2009)

EAS	rs16972959	23901376	16p11.2	1.35×10^{-09}	(Sheng et al. 2011)
EAS	rs12599402	11189888	16p13	1.34×10^{-8}	(Zhang et al. 2011)
EAS	rs8016947	35832666	14q13	1.08×10^{-13}	(Li et al. 2013)
EAS	rs4649203	24519920	1p36	9.90×10^{-9}	(Li et al. 2013)
EAS	rs6705628	74208362	2p13	6.9×10^{-17}	(Yang et al. 2013)
EAS	rs4852324	74202578	2p13	5.7×10^{-14}	(Yang et al. 2013)
EAS	rs6804441	119260944	3q13	2.5×10^{-16}	(Yang et al. 2013)
EAS	rs4948496	63805617	10q21	5.1×10^{-11}	(Yang et al. 2013)
EAS	rs12822507	12773521	12p13	2.2×10^{-8}	(Yang et al. 2013)
EAS	rs10845606	12834894	12p13	3.8×10^{-17}	(Yang et al. 2013)
EAS	rs34330	12870695	12p13	4.8×10^{-12}	(Yang et al. 2013)
EAS	rs4622329	102321935	12p23	9.4×10^{-12}	(Yang et al. 2013)
EAS	rs906868	30448344	2p23.1	7.71×10^{-10}	(Yu et al. 2013)
EAS	rs7579944	30445026	2p23.1	5.55×10^{-9}	(Yu et al. 2013)
EAS	rs11603023	118486067	11q23.3	1.25×10^{-08}	(Zhang et al. 2014)
EAS	rs10892301	118735476	11q23.3	2.51×10^{-08}	(Zhang et al. 2014)
EAS	rs7601754	191940451	2q32.3	1.39×10^{-9}	(Yang et al. 2009)
EAS	rs17266594	102750922	4q24	4.67×10^{-9}	(Chang et al. 2009)
EAS	rs1128334	128328959	11q24.3	2.33×10^{-11}	(Yang et al. 2010)
EAS	rs7097397	50025396	10q11.23	8.15×10^{-12}	(Yang et al. 2010)
EAS	rs10168266	191935804	2q32.3	2.17×10^{-8}	(Yang et al. 2010)
EAS	rs9271366	32586854	6p21.32	7.67×10^{-10}	(Yang et al. 2010)
EAS	rs7329174	41558110	13q14.11	1.47×10^{-8}	(Yang et al. 2011)
EAS	rs548234	106568034	6q21	1.28×10^{-16}	(Zhou et al. 2011)
EAS	rs2222631	119272391	3q13.33	4.50×10^{-8}	(Zhang et al. 2015c)
EAS	rs2298428	21982892	22q11.21	3.16×10^{-9}	(Zhang et al. 2015b)

EAS	rs1418190	173361979	1q25.1	1.08×10^{-08}	(Sheng et al. 2015)
EAS	rs4916219	173373183	1q25	7.77×10^{-09}	(Sheng et al. 2015)
EAS	rs2934498	85968282	16q24.1	4.97×10^{-09}	(Sheng et al. 2015)
EAS	rs2431697	159879978	5q34	1.15×10^{-22}	(Sheng et al. 2015)
EAS	rs2732547	35088683	11p13	1.55×10^{-11}	(Sheng et al. 2015)
EAS	rs12494314	119122820	3q13.33	1.01×10^{-09}	(Sheng et al. 2015)
EAS	rs13205210	34831856	6p21.31	5.51×10^{-12}	(Zhang et al. 2015a)
EAS	rs10036748	150458146	5q33.1	1.94×10^{-10}	(Zhang et al. 2015a)
EAS	rs73366469	74033600	7q11.23	3.75×10^{-117}	(Sun et al. 2016)
EAS	rs10807150	35272274	6p21.31	6.06×10^{-16}	(Sun et al. 2016)
EAS	rs2421184	158886939	5q33.3	4.67×10^{-12}	(Sun et al. 2016)
EAS	rs7726414	133431834	5q31.1	1.13×10^{-11}	(Sun et al. 2016)
EAS	rs7726159	1282319	5p15.33	2.11×10^{-11}	(Sun et al. 2016)
EAS	rs1610555	67543147	18q22.2	4.50×10^{-11}	(Sun et al. 2016)
EAS	rs2009453	65399528	11q13.1	9.61×10^{-11}	(Sun et al. 2016)
EAS	rs12900339	38927386	15q14	4.73×10^{-10}	(Sun et al. 2016)
EAS	rs61616683	39755773	22q13.1	5.73×10^{-10}	(Sun et al. 2016)
EAS	rs2305772	52033742	19q13.41	1.34×10^{-9}	(Sun et al. 2016)
CEU	rs13277113	11349186	8p21.3	1×10^{-10}	(Hom et al. 2008)
CEU	rs11574637	31368874	16p11.2	3×10^{-11}	(Hom et al. 2008)
CEU	rs3131379	31721033	6p21.33	1.71×10^{-52}	(International Consortium for Systemic Lupus Erythematosus et al. 2008)
CEU	rs9888739	31313253	16pll.2	1.61×10^{-23}	(International Consortium for Systemic Lupus Erythematosus et al. 2008)
CEU	rs1143678	31343005	16pll.2	8.5×10^{-14}	(International Consortium for Systemic Lupus Erythematosus et al. 2008)
CEU	rs4548893	31364493	16pll.2	2.36×10^{-12}	(International Consortium for Systemic Lupus Erythematosus et al. 2008)
CEU	rs729302	128568960	7q32.1	2×10^{-10}	(International Consortium for Systemic Lupus Erythematosus et al. 2008)
CEU	rsl0279821	128683547	7q32.1	6.5×10^{-9}	(International Consortium for Systemic Lupus Erythematosus et al. 2008)

CEU	rsl2537284	128717906	7q32.1	3.61×10^{-19}	(International Consortium for Systemic Lupus Erythematosus et al. 2008)
CEU	rs4963128	589564	llpl5.5	3.00×10^{-10}	(International Consortium for Systemic Lupus Erythematosus et al. 2008)
CEU	rs6445975	58370177	3pl4.3	7.10×10^{-09}	(International Consortium for Systemic Lupus Erythematosus et al. 2008)
CEU	rs10239340	128668510	7q32.1	6.98×10^{-16}	(International Consortium for Systemic Lupus Erythematosus et al. 2008)
CEU	rs6985109	10761585	8p23.1	2.51×10^{-11}	(International Consortium for Systemic Lupus Erythematosus et al. 2008)
CEU	rs4240671	10767748	8p23.1	6.6×10^{-9}	(International Consortium for Systemic Lupus Erythematosus et al. 2008)
CEU	rs11783247	10788875	8p23.1	8.00×10^{-10}	(International Consortium for Systemic Lupus Erythematosus et al. 2008)
CEU	rs6984496	10796093	8p23.1	2.00×10^{-10}	(International Consortium for Systemic Lupus Erythematosus et al. 2008)
CEU	rs7836059	11272164	8p23.1	4×10^{-10}	(International Consortium for Systemic Lupus Erythematosus et al. 2008)
CEU	rs2248932	11391650	8p23.1	7×10^{-10}	(International Consortium for Systemic Lupus Erythematosus et al. 2008)
CEU	rs7829816	56849386	8q12.1	5.4×10^{-9}	(International Consortium for Systemic Lupus Erythematosus et al. 2008)
CEU	rs2667978	56897951	8q12.1	5.1×10^{-8}	(International Consortium for Systemic Lupus Erythematosus et al. 2008)
CEU	rs5754217	21939675	22q11.21	7.53×10^{-8}	(International Consortium for Systemic Lupus Erythematosus et al. 2008)
CEU	rs7775397	32261252	6p21.32	8.0×10^{-47}	(International Consortium for Systemic Lupus Erythematosus et al. 2008)
CEU	rs9275572	32678999	6p21.32	7.0×10^{-48}	(International Consortium for Systemic Lupus Erythematosus et al. 2008)
CEU	rs1794282	32666526	6p21.32	2.6×10^{-46}	(International Consortium for Systemic Lupus Erythematosus et al. 2008)
CEU	rs7192	32411646	6p21.32	6.2×10^{-40}	(International Consortium for Systemic Lupus Erythematosus et al. 2008)
CEU	rs10516487	102751076	4q24	3.74×10^{-10}	(Kozyrev et al. 2008)
CEU	rs17266594	102750922	4q24	4.74×10^{-11}	(Kozyrev et al. 2008)
CEU	rs5029939	138195723	6q23.3	2.89×10^{-12}	(Graham et al. 2008)
CEU	rs3821236	191902758	2q32.3	8.49×10^{-11}	(Graham et al. 2008)
CEU	rs2618476	11352541	8p23.1	1.70×10^{-8}	(Graham et al. 2008)
CEU	rs3135394	32408497	6p21.32	2.0×10^{-60}	(Gateva et al. 2009)
CEU	rs7574865	191964633	2q32.3	1.4×10^{-41}	(Gateva et al. 2009)
CEU	rs2070197	128589000	7q32.1	5.8×10^{-24}	(Gateva et al. 2009)
CEU	rs11860650	31326706	16p11.2	1.9×10^{-20}	(Gateva et al. 2009)

CEU	rs5029937	138195151	6q23.3	5.3×10^{-13}	(Gateva et al. 2009)
CEU	rs2205960	173191475	1q25.1	6.3×10^{-9}	(Gateva et al. 2009)
CEU	rs7708392	150457485	5q33.1	3.8×10^{-13}	(Gateva et al. 2009)
CEU	rs11755393	34824636	6p21.31	2.2×10^{-8}	(Gateva et al. 2009)
CEU	rs2732552	35084592	11p13	1.82×10^{-9}	(Lessard et al. 2011)
CEU	rs387619	35098193	11p13	1.46×10^{-8}	(Lessard et al. 2011)
CEU	rs2301271	32725193	6p21.32	2.00×10^{-12}	(Chung et al. 2011)
CEU	rs10911363	183549757	1q25.3	2.87×10^{-11}	(Cunninghame Graham et al. 2011)
CEU	rs2366293	50227828	7p12.2	2.33×10^{-9}	(Cunninghame Graham et al. 2011)
CEU	rs2280381	86018633	16q24.1	1.24×10^{-8}	(Cunninghame Graham et al. 2011)
CEU	rs1990760	163124051	2q24.2	1.63×10^{-8}	(Cunninghame Graham et al. 2011)
CEU	rs280519	10472933	19p13.2	3.88×10^{-8}	(Cunninghame Graham et al. 2011)
CEU	rs1269852	32080191	6p21.33	2.48×10^{-19}	(Fernando et al. 2012)
CEU	rs3906272	31262924	6p21.33	2.95×10^{-10}	(Fernando et al. 2012)
CEU	rs11648084	85972598	16q24.1	2.34×10^{-9}	(Lessard et al. 2012)
CEU	rs9936079	85967594	16q24.1	3.96×10^{-9}	(Lessard et al. 2012)
CEU	rs4843865	85969305	16q24.1	2.93×10^{-8}	(Lessard et al. 2012)
CEU	rs11347703	85969641	16q24.1	1.11×10^{-8}	(Lessard et al. 2012)
CEU	rs11117422	85972013	16q24.1	9.37×10^{-9}	(Lessard et al. 2012)
CEU	rs13335265	85972810	16q24.1	1.23×10^{-8}	(Lessard et al. 2012)
CEU	rs12711490	85973028	16q24.1	2.11×10^{-8}	(Lessard et al. 2012)
CEU	rs11641153	85973140	16q24.1	1.31×10^{-9}	(Lessard et al. 2012)
CEU	rs11641155	85973152	16q24.1	1.23×10^{-9}	(Lessard et al. 2012)
CEU	rs7205434	85973195	16q24.1	1.23×10^{-9}	(Lessard et al. 2012)
CEU	rs4843868	85973401	16q24.1	8.57×10^{-10}	(Lessard et al. 2012)
CEU	rs4843323	85974961	16q24.1	7.71×10^{-10}	(Lessard et al. 2012)

CEU	rs4843869	85975141	16q24.1	7.61×10^{-10}	(Lessard et al. 2012)
CEU	rs7202472	85977502	16q24.1	7.25×10^{-9}	(Lessard et al. 2012)
CEU	rs2051549	32730086	6p21.32	3.36×10^{-22}	(Lee et al. 2012)
CEU	rs12531711	128617466	7q32.1	6.41×10^{-13}	(Lee et al. 2012)
CEU	rs12680762	11332026	8p23.1	1.45×10^{-8}	(Lee et al. 2012)
CEU	rs10931481	191954852	2q32.3	1.74×10^{-8}	(Lee et al. 2012)
CEU	rs2275247	35908451	1p34.3	1.4×10^{-10}	(Martin et al. 2013)
CEU	rs2176082	58331186	3p14.3	5.24×10^{-11}	(Martin et al. 2013)
CEU	rs1635852	28189411	7p15.1	1.6×10^{-8}	(Martin et al. 2013)
CEU	rs10911628	184649503	1q25.3	2.3×10^{-13}	(Armstrong et al. 2014)
CEU	rs4728142	128573967	7q32.1	7.1×10^{-10}	(Armstrong et al. 2014)
CEU	rs11073328	38764843	15q14	9.9×10^{-15}	(Armstrong et al. 2014)
CEU	rs8023715	97607681	15q26.2	1.2×10^{-8}	(Armstrong et al. 2014)
CEU	rs11697848	48575315	20q13.13	1.4×10^{-11}	(Armstrong et al. 2014)
CEU	rs7897633	52957721	10q21.1	2.75×10^{-8}	(Kariuki et al. 2015)
CEU	rs6445972	58321707	3p14.3	4.62×10^{-10}	(Vaughn et al. 2014)
CEU	rs10506216	43130885	12q12	3.08×10^{-8}	(Demirci et al. 2015)
CEU	rs1143679	31276811	16p11.2	1.1×10^{-16}	(Suarez-Gestal et al. 2009)
CEU	rs2476601	114377568	1p13.2	1.10×10^{-28}	(Bentham et al. 2015)
CEU	rs1801274	161479745	1q23.3	1.04×10^{-12}	(Bentham et al. 2015)
CEU	rs704840	173226195	1q25.1	3.12×10^{-19}	(Bentham et al. 2015)
CEU	rs17849501	183542323	1q25.3	3.45×10^{-88}	(Bentham et al. 2015)
CEU	rs3024505	206939904	1q32.1	4.64×10^{-9}	(Bentham et al. 2015)
CEU	rs9782955	236039877	1q42.3	1.25×10^{-9}	(Bentham et al. 2015)
CEU	rs2111485	163110536	2q24.2	1.27×10^{-11}	(Bentham et al. 2015)

CEU	rs11889341	191943742	2q32.3	5.59×10^{-122}	(Bentham et al. 2015)
CEU	rs3768792	213871709	2q34	1.21×10^{-13}	(Bentham et al. 2015)
CEU	rs9311676	58470351	3p14.3	3.06×10^{-14}	(Bentham et al. 2015)
CEU	rs564799	159728987	3q25.33	1.54×10^{-9}	(Bentham et al. 2015)
CEU	rs10028805	102737250	4q24	4.31×10^{-17}	(Bentham et al. 2015)
CEU	rs7726414	133431834	5q31.1	4.44×10^{-16}	(Bentham et al. 2015)
CEU	rs10036748	150458146	5q33.1	1.27×10^{-45}	(Bentham et al. 2015)
CEU	rs2431697	159879978	5q33.3	8.01×10^{-28}	(Bentham et al. 2015)
CEU	rs1270942	31918860	6p21.33	2.25×10^{-165}	(Bentham et al. 2015)
CEU	rs9462027	34797241	6p21.31	7.55×10^{-9}	(Bentham et al. 2015)
CEU	rs6568431	106588806	6q21	5.04×10^{-14}	(Bentham et al. 2015)
CEU	rs6932056	138242437	6q23.3	1.97×10^{-31}	(Bentham et al. 2015)
CEU	rs849142	28185891	7p15.1	8.61×10^{-11}	(Bentham et al. 2015)
CEU	rs4917014	50305863	7p12.2	6.39×10^{-14}	(Bentham et al. 2015)
CEU	rs10488631	128594183	7q32.1	9.37×10^{-110}	(Bentham et al. 2015)
CEU	rs2736340	11343973	8p23.1	6.28×10^{-20}	(Bentham et al. 2015)
CEU	rs2663052	50069395	10q11.23	5.25×10^{-9}	(Bentham et al. 2015)
CEU	rs4948496	63805617	10q21.2	1.04×10^{-10}	(Bentham et al. 2015)
CEU	rs12802200	566936	11p15.5	8.81×10^{-10}	(Bentham et al. 2015)
CEU	rs2732549	35088399	11p13	1.20×10^{-23}	(Bentham et al. 2015)
CEU	rs3794060	71187679	11q13.4	1.32×10^{-20}	(Bentham et al. 2015)
CEU	rs7941765	128499000	11q24.3	1.35×10^{-10}	(Bentham et al. 2015)
CEU	rs10774625	111910219	12q24.12	4.09×10^{-9}	(Bentham et al. 2015)
CEU	rs1059312	129278864	12q24.32	1.48×10^{-13}	(Bentham et al. 2015)
CEU	rs4902562	68731458	14q24.1	6.15×10^{-10}	(Bentham et al. 2015)
CEU	rs2289583	75311036	15q24.2	6.22×10^{-15}	(Bentham et al. 2015)
CEU	rs9652601	11174365	16p13.13	7.42×10^{-17}	(Bentham et al. 2015)

CEU	rs34572943	31272353	16p11.2	3.39×10^{-76}	(Bentham et al. 2015)
CEU	rs11644034	85972612	16q24.1	9.58×10^{-18}	(Bentham et al. 2015)
CEU	rs2286672	4712617	17p13.2	2.93×10^{-9}	(Bentham et al. 2015)
CEU	rs2941509	37921194	17q12	7.98×10^{-9}	(Bentham et al. 2015)
CEU	rs2304256	10475652	19p13.2	3.50×10^{-13}	(Bentham et al. 2015)
CEU	rs7444	21976934	22q11.21	1.84×10^{-22}	(Bentham et al. 2015)

References

Armstrong, D.L., R. Zidovetzki, M.E. Alarcon-Riquelme, B.P. Tsao, L.A. Criswell *et al.*, 2014 GWAS identifies novel SLE susceptibility genes and explains the association of the HLA region. *Genes Immun* 15 (6):347-354.

Bentham, J., D.L. Morris, D.S. Cunninghame Graham, C.L. Pinder, P. Tombleson *et al.*, 2015 Genetic association analyses implicate aberrant regulation of innate and adaptive immunity genes in the pathogenesis of systemic lupus erythematosus. *Nat Genet*.

Chang, Y.K., W. Yang, M. Zhao, C.C. Mok, T.M. Chan *et al.*, 2009 Association of BANK1 and TNFSF4 with systemic lupus erythematosus in Hong Kong Chinese. *Genes Immun* 10 (5):414-420.

Chung, S.A., K.E. Taylor, R.R. Graham, J. Nititham, A.T. Lee *et al.*, 2011 Differential genetic associations for systemic lupus erythematosus based on anti-dsDNA autoantibody production. *PLoS Genet* 7 (3):e1001323.

Cunninghame Graham, D.S., D.L. Morris, T.R. Bhangale, L.A. Criswell, A.C. Syvanen *et al.*, 2011 Association of NCF2, IKZF1, IRF8, IFIH1, and TYK2 with systemic lupus erythematosus. *PLoS Genet* 7 (10):e1002341.

Demirci, F.Y., X. Wang, J.A. Kelly, D.L. Morris, M.M. Barmada *et al.*, 2015 Identification of a New Susceptibility Locus for Systemic Lupus Erythematosus on Chromosome 12 in Individuals of European Ancestry. *Arthritis Rheumatol*.

Fernando, M.M., J. Freudenberg, A. Lee, D.L. Morris, L. Boteva *et al.*, 2012 Transancestral mapping of the MHC region in systemic lupus erythematosus identifies new independent and interacting loci at MSH5, HLA-DPB1 and HLA-G. *Ann Rheum Dis* 71 (5):777-784.

Gateva, V., J.K. Sandling, G. Hom, K.E. Taylor, S.A. Chung *et al.*, 2009 A large-scale replication study identifies TNIP1, PRDM1, JAZF1, UHRF1BP1 and IL10 as risk loci for systemic lupus erythematosus. *Nat Genet* 41 (11):1228-1233.

Graham, R.R., C. Cotsapas, L. Davies, R. Hackett, C.J. Lessard *et al.*, 2008 Genetic variants near TNFAIP3 on 6q23 are associated with systemic lupus erythematosus. *Nat Genet* 40 (9):1059-1061.

Han, J.W., H.F. Zheng, Y. Cui, L.D. Sun, D.Q. Ye *et al.*, 2009 Genome-wide association study in a Chinese Han population identifies nine new susceptibility loci for systemic lupus erythematosus. *Nat Genet* 41 (11):1234-1237.

Hom, G., R.R. Graham, B. Modrek, K.E. Taylor, W. Ortmann *et al.*, 2008 Association of systemic lupus erythematosus with C8orf13-BLK and ITGAM-ITGAX. *N Engl J Med* 358 (9):900-909.

International Consortium for Systemic Lupus Erythematosus, G., J.B. Harley, M.E. Alarcon-Riquelme, L.A. Criswell, C.O. Jacob *et al.*, 2008 Genome-wide association scan in women with systemic lupus erythematosus identifies susceptibility variants in ITGAM, PXK, KIAA1542 and other loci. *Nat Genet* 40 (2):204-210.

Kariuki, S.N., Y. Ghodke-Puranik, J.M. Dorschner, B.S. Chrabot, J.A. Kelly *et al.*, 2015 Genetic analysis of the pathogenic molecular sub-phenotype interferon-alpha identifies multiple novel loci involved in systemic lupus erythematosus. *Genes Immun* 16 (1):15-23.

Kozyrev, S.V., A.K. Abelson, J. Wojcik, A. Zaghlool, M.V. Linga Reddy *et al.*, 2008 Functional variants in the B-cell gene BANK1 are associated with systemic lupus erythematosus. *Nat Genet* 40 (2):211-216.

Lee, Y.H., S.C. Bae, S.J. Choi, J.D. Ji, and G.G. Song, 2012 Genome-wide pathway analysis of genome-wide association studies on systemic lupus erythematosus and rheumatoid arthritis. *Mol Biol Rep* 39 (12):10627-10635.

Lessard, C.J., I. Adrianto, J.A. Ice, G.B. Wiley, J.A. Kelly *et al.*, 2012 Identification of IRF8, TMEM39A, and IKZF3-ZPBP2 as susceptibility loci for systemic lupus erythematosus in a large-scale multiracial replication study. *Am J Hum Genet* 90 (4):648-660.

Lessard, C.J., I. Adrianto, J.A. Kelly, K.M. Kaufman, K.M. Grundahl *et al.*, 2011 Identification of a systemic lupus erythematosus susceptibility locus at 11p13 between PDHX and CD44 in a multiethnic study. *Am J Hum Genet* 88 (1):83-91.

Li, Y., H. Cheng, X.B. Zuo, Y.J. Sheng, F.S. Zhou *et al.*, 2013 Association analyses identifying two common susceptibility loci shared by psoriasis and systemic lupus erythematosus in the Chinese Han population. *J Med Genet* 50 (12):812-818.

Martin, J.E., S. Assassi, L.M. Diaz-Gallo, J.C. Broen, C.P. Simeon *et al.*, 2013 A systemic sclerosis and systemic lupus erythematosus pan-meta-GWAS reveals new shared susceptibility loci. *Hum Mol Genet* 22 (19):4021-4029.

Sheng, Y.J., J.P. Gao, J. Li, J.W. Han, Q. Xu *et al.*, 2011 Follow-up study identifies two novel susceptibility loci PRKCB and 8p11.21 for systemic lupus erythematosus. *Rheumatology (Oxford)* 50 (4):682-688.

Sheng, Y.J., J.H. Xu, Y.G. Wu, X.B. Zuo, J.P. Gao *et al.*, 2015 Association analyses confirm five susceptibility loci for systemic lupus erythematosus in the Han Chinese population. *Arthritis Res Ther* 17:85.

Suarez-Gestal, M., M. Calaza, E. Endreffy, R. Pullmann, J. Ordi-Ros *et al.*, 2009 Replication of recently identified systemic lupus erythematosus genetic associations: a case-control study. *Arthritis Res Ther* 11 (3):R69.

Sun, C., J.E. Molineros, L.L. Looger, X.J. Zhou, K. Kim *et al.*, 2016 High-density genotyping of immune-related loci identifies new SLE risk variants in individuals with Asian ancestry. *Nat Genet* 48 (3):323-330.

Vaughn, S.E., C. Foley, X. Lu, Z.H. Patel, E.E. Zoller *et al.*, 2014 Lupus risk variants in the PXK locus alter B-cell receptor internalization. *Front Genet* 5:450.

Yang, J., W. Yang, N. Hirankarn, D.Q. Ye, Y. Zhang *et al.*, 2011 ELF1 is associated with systemic lupus erythematosus in Asian populations. *Hum Mol Genet* 20 (3):601-607.

Yang, W., P. Ng, M. Zhao, N. Hirankarn, C.S. Lau *et al.*, 2009 Population differences in SLE susceptibility genes: STAT4 and BLK, but not PXK, are associated with systemic lupus erythematosus in Hong Kong Chinese. *Genes Immun* 10 (3):219-226.

Yang, W., N. Shen, D.Q. Ye, Q. Liu, Y. Zhang *et al.*, 2010 Genome-wide association study in Asian populations identifies variants in ETS1 and WDFY4 associated with systemic lupus erythematosus. *PLoS Genet* 6 (2):e1000841.

Yang, W., H. Tang, Y. Zhang, X. Tang, J. Zhang *et al.*, 2013 Meta-analysis followed by replication identifies loci in or near CDKN1B, TET3, CD80, DRAM1, and ARID5B as associated with systemic lupus erythematosus in Asians. *Am J Hum Genet* 92 (1):41-51.

Yu, Z.Y., W.S. Lu, X.B. Zuo, J. Hu, S. Yao *et al.*, 2013 One novel susceptibility locus associate with systemic lupus erythematosus in Chinese Han population. *Rheumatol Int* 33 (8):2079-2083.

Zhang, J., L. Zhang, Y. Zhang, J. Yang, M. Guo *et al.*, 2015a Gene-Based Meta-Analysis of Genome-Wide Association Study Data Identifies Independent Single-Nucleotide Polymorphisms in ANXA6 as Being Associated With Systemic Lupus Erythematosus in Asian Populations. *Arthritis Rheumatol* 67 (11):2966-2977.

Zhang, J., Y. Zhang, J. Yang, L. Zhang, L. Sun *et al.*, 2014 Three SNPs in chromosome 11q23.3 are independently associated with systemic lupus erythematosus in Asians. *Hum Mol Genet* 23 (2):524-533.

Zhang, Y., Y.F. Wang, J. Yang, J. Zhang, L. Sun *et al.*, 2015b Meta-analysis of two Chinese populations identifies an autoimmune disease risk allele in 22q11.21 as associated with systemic lupus erythematosus. *Arthritis Res Ther* 17:67.

Zhang, Y., J. Yang, J. Zhang, L. Sun, N. Hirankarn *et al.*, 2015c Genome-wide search followed by replication reveals genetic interaction of CD80 and ALOX5AP associated with systemic lupus erythematosus in Asian

populations. *Ann Rheum Dis*.
Zhang, Z., Y. Cheng, X. Zhou, Y. Li, J. Gao *et al.*, 2011 Polymorphisms at 16p13 are associated with systemic lupus erythematosus in the Chinese population. *J Med Genet* 48 (1):69-72.
Zhou, X.J., X.L. Lu, J.C. Lv, H.Z. Yang, L.X. Qin *et al.*, 2011 Genetic association of PRDM1-ATG5 intergenic region and autophagy with systemic lupus erythematosus in a Chinese population. *Ann Rheum Dis* 70 (7):1330-1337.
