# Supplementary material for: Several Critical Cell Types, Tissues, and Pathways Are Implicated in Genome-Wide Association Studies for Systemic Lupus Erythematosus
Source: G3 (Bethesda). 2016 Mar 23;6(6):1503–11. doi: 10.1534/g3.116.027326 (PMC4889647; doi:10.1534/g3.116.027326)
Supplement: Supplemental Material [file supp_g3.116.027326_TableS2.pdf]

Table S2. The cell enrichment of SLE implicated genes within 533 cell types expression matrix in Homo sapiens. *The cells filled in yellow mean passing the Bonferroni-corrected significance criteria ($P \leq 9.38 \times 10^{-5}$).*

Cell	Eastern Asian	Caucasian	Caucasian without HLA region
Burkitt's lymphoma cell line:DAUDI.CNhs10739.10422-106C8	8.55E-05	6.67E-03	6.82E-03
CD14+ Monocytes	7.33E-05	2.74E-04	1.27E-04
CD14+ monocytes - treated with BCG	4.76E-05	2.05E-03	1.14E-03
CD14+ monocytes - treated with Group A streptococci	2.30E-05	2.00E-05	9.77E-05
CD14+ monocytes - treated with IFN + N-hexane	8.55E-05	4.20E-04	2.64E-04
CD14+ monocytes - treated with Salmonella	1.00E-06	1.00E-06	1.00E-06
CD14+ monocytes - treated with lipopolysaccharide	6.00E-06	9.00E-06	7.00E-06
CD19+ B Cells	1.00E-06	2.00E-06	1.00E-06
CD19+ B Cells (pluriselect)	3.00E-06	2.00E-05	2.30E-05
CD4+ T Cells	8.79E-05	3.31E-03	2.76E-03
CD4+CD25+CD45RA- memory regulatory T cells	2.20E-05	3.42E-04	7.83E-04
CD4+CD25+CD45RA- memory regulatory T cells expanded	8.55E-05	5.08E-03	8.71E-03
CD4+CD25-CD45RA- memory conventional T cells	5.00E-06	2.20E-02	1.10E-02
Dendritic Cells - plasmacytoid	9.00E-06	5.00E-06	2.00E-06
Eosinophils	1.06E-03	1.20E-05	1.60E-05
Natural Killer Cells	3.42E-05	1.10E-04	1.42E-04
Neutrophils	1.55E-02	2.00E-06	3.00E-06
Peripheral Blood Mononuclear Cells	2.00E-06	1.61E-04	6.10E-05
b cell line:RPMI1788.CNhs10744.10427-106D4	1.00E-06	1.81E-04	3.42E-04
blood	7.57E-05	1.25E-03	9.39E-04
diffuse large B-cell lymphoma cell	1.10E-05	6.67E-03	7.30E-03

line:CTB-1.CNhs11741.10631-108I1			
migratory langerhans cells	2.20E-05	1.71E-04	1.32E-04
Plasma cell leukemia cell line:ARH-77.CNhs12807.10840-111E3	2.00E-06	1.00E-06	1.00E-06
lymph node	3.42E-05	1.03E-02	9.20E-03
lymphoma	8.79E-05	1.15E-04	2.74E-04
onsil	3.54E-05	3.62E-04	3.52E-04
splenic lymphoma with villous lymphocytes cell line:SLVL.CNhs10741.10424-106D1	4.27E-05	5.48E-04	3.42E-04
xeroderma pigentosum b cell line:XPL 17.CNhs11813.10563-108A5	1.00E-06	1.00E-06	1.00E-06
mycosis fungoides	6.00E-06	2.10E-04	6.26E-04
