# Supplementary material for: Several Critical Cell Types, Tissues, and Pathways Are Implicated in Genome-Wide Association Studies for Systemic Lupus Erythematosus
Source: G3 (Bethesda). 2016 Mar 23;6(6):1503–11. doi: 10.1534/g3.116.027326 (PMC4889647; doi:10.1534/g3.116.027326)
Supplement: Supplemental Material [file supp_g3.116.027326_TableS3.pdf]

Table S3. The cell enrichment of SLE implicated genes within 249 cell types expression matrix for mus musculus. *The cells filled in yellow mean the P values passing the Bonferroni-corrected significance criteria ($P \le 2.01 \times 10^{-4}$).*

Condition	Eastern Asian	Caucasian	Caucasian without HLA region
B.T1.Sp	4.88E-05	5.68E-04	1.86E-04
B.T2.Sp	6.00E-06	6.23E-05	2.80E-05
B.T3.Sp	7.00E-06	2.90E-05	1.50E-05
B.Fo.Sp	5.00E-06	5.08E-04	1.91E-04
B.MZ.Sp	2.50E-05	6.26E-04	3.32E-04
B1a.Sp	3.00E-05	3.42E-04	2.74E-04
B.FrF.BM	7.00E-06	2.99E-03	2.13E-03
B.Fo.MLN	1.50E-05	6.07E-04	1.71E-04
B.Fo.LN	7.00E-06	4.20E-04	1.61E-04
B.Fo.PC	3.42E-05	2.83E-03	1.33E-03
B1a.PC	1.00E-04	4.44E-03	3.70E-03
CD19Control	5.00E-06	1.29E-04	6.59E-05
