# Supplementary material for: Several Critical Cell Types, Tissues, and Pathways Are Implicated in Genome-Wide Association Studies for Systemic Lupus Erythematosus
Source: G3 (Bethesda). 2016 Mar 23;6(6):1503–11. doi: 10.1534/g3.116.027326 (PMC4889647; doi:10.1534/g3.116.027326)
Supplement: Supplemental Material [file supp_g3.116.027326_TableS4.pdf]

Table S4. The tissue enrichment of SLE implicated genes within 79 tissues expression matrix in homo-sapiens. *The cells filled in yellow mean the P values passing the Bonferroni-corrected significance criteria ($P \leq 6.33 \times 10^{-4}$).*

Cells	Eastern Asian	Caucasian	Caucasian without HLA region
Whole_Blood	3.78E-03	3.30E-05	4.15E-05
PB-BDCA4+Dentritic_cells	7.63E-04	3.00E-06	1.00E-06
PB-CD56+NK_cells	8.30E-05	3.62E-04	5.09E-04
PB-CD4+T_cells	7.57E-05	8.38E-03	6.82E-03
PB-CD8+T_cells	3.32E-04	6.98E-03	3.94E-03
PB-CD19+B_cells	1.00E-06	1.37E-03	3.31E-03
