# Supplementary material for: Several Critical Cell Types, Tissues, and Pathways Are Implicated in Genome-Wide Association Studies for Systemic Lupus Erythematosus
Source: G3 (Bethesda). 2016 Mar 23;6(6):1503–11. doi: 10.1534/g3.116.027326 (PMC4889647; doi:10.1534/g3.116.027326)
Supplement: Supplemental Material [file supp_g3.116.027326_TableS5.pdf]

Table S5. The pathway enrichment of SLE implicated genes within 1,751 Gene Expression Ontology matrix. *The cells filled in yellow mean the P values passing the Bonferroni-corrected significance criteria ($P \leq 2.86 \times 10^{-5}$).*

GO Pathway	Eastern Asian	Caucasian	Caucasian without HLA region
GO:0001816 cytokine production	2.20E-02	5.00E-06	3.00E-06
GO:0001817 regulation of cytokine production	6.07E-04	3.00E-06	1.00E-06
GO:0001818 negative regulation of cytokine production	2.68E-03	8.00E-06	8.00E-06
GO:0002237 response to molecule of bacterial origin	5.87E-03	2.00E-06	2.00E-06
GO:0002252 immune effector process	3.31E-03	5.00E-06	1.00E-06
GO:0002253 activation of immune response	1.49E-03	5.00E-06	1.10E-05
GO:0002263 cell activation involved in immune response	1.29E-02	7.00E-06	3.00E-06
GO:0002366 leukocyte activation involved in immune response	1.19E-02	7.00E-06	3.00E-06
GO:0002443 leukocyte mediated immunity	1.79E-01	4.00E-06	5.00E-06
GO:0002684 positive regulation of immune system process	1.60E-05	2.00E-06	1.00E-06
GO:0002694 regulation of leukocyte activation	1.69E-03	1.00E-06	1.00E-06
GO:0002695 negative regulation of leukocyte activation	1.02E-03	1.00E-06	2.00E-06
GO:0002696 positive regulation of leukocyte activation	2.20E-03	1.40E-05	1.40E-05
GO:0002697 regulation of immune effector process	1.42E-04	7.00E-06	5.00E-06
GO:0002699 positive regulation of immune effector process	1.14E-03	3.00E-06	4.00E-06
GO:0002703 regulation of leukocyte mediated immunity	8.09E-03	1.00E-06	4.00E-06
GO:0002757 immune response-activating signal transduction	6.85E-04	5.00E-06	9.00E-06
GO:0002764 immune response-regulating signaling pathway	2.64E-04	9.00E-06	1.00E-06
GO:0002819 regulation of adaptive immune response	4.03E-05	1.00E-06	1.00E-06
GO:0002822 regulation of adaptive immune response based on somatic recombination of immune receptors built from immunoglobulin superfamily domains	3.17E-05	4.00E-06	5.00E-06
GO:0006954 inflammatory response	1.35E-02	2.10E-05	5.00E-06
GO:0009617 response to bacterium	2.86E-02	7.00E-06	2.00E-06
GO:0031347 regulation of defense response	3.52E-04	3.00E-06	7.00E-06

GO:0031349 positive regulation of defense response	2.30E-04	3.30E-05	2.60E-05
GO:0032496 response to lipopolysaccharide	4.40E-02	6.00E-06	5.00E-06
GO:0032944 regulation of mononuclear cell proliferation	5.24E-03	5.00E-06	3.00E-06
GO:0045087 innate immune response	3.13E-02	1.20E-05	5.00E-06
GO:0045321 leukocyte activation	2.39E-03	1.00E-06	2.00E-06
GO:0050670 regulation of lymphocyte proliferation	6.82E-03	4.00E-06	2.00E-06
GO:0050776 regulation of immune response	9.28E-05	1.00E-06	1.00E-06
GO:0050778 positive regulation of immune response	4.40E-05	2.00E-06	1.00E-06
GO:0050865 regulation of cell activation	2.28E-03	1.00E-06	1.00E-06
GO:0050866 negative regulation of cell activation	1.29E-03	2.00E-06	2.00E-06
GO:0050867 positive regulation of cell activation	2.60E-03	1.90E-05	2.80E-05
GO:0051249 regulation of lymphocyte activation	6.19E-03	1.00E-06	1.00E-06
GO:0051250 negative regulation of lymphocyte activation	7.04E-04	1.00E-06	1.00E-06
GO:0051707 response to other organism	3.99E-02	5.62E-05	2.40E-05
GO:0070663 regulation of leukocyte proliferation	6.06E-04	1.00E-06	1.00E-06
GO:0071219 cellular response to molecule of bacterial origin	5.71E-03	1.50E-05	2.10E-05
GO:0071222 cellular response to lipopolysaccharide	4.57E-03	9.00E-06	1.20E-05
GO:1902105 regulation of leukocyte differentiation	1.87E-02	5.00E-06	5.00E-06
